# Estimating undetected Ebola spillovers

**DOI:** 10.1371/journal.pntd.0007428

**Published:** 2019-06-13

**Authors:** Emma E. Glennon, Freya L. Jephcott, Olivier Restif, James L. N. Wood

**Affiliations:** Department of Veterinary Medicine, University of Cambridge, Cambridge United Kingdom; Institute for Disease Modeling, UNITED STATES

## Abstract

The preparedness of health systems to detect, treat, and prevent onward transmission of Ebola virus disease (EVD) is central to mitigating future outbreaks. Early detection of outbreaks is critical to timely response, but estimating detection rates is difficult because unreported spillover events and outbreaks do not generate data. Using three independent datasets available on the distributions of secondary infections during EVD outbreaks across West Africa, in a single district (Western Area) of Sierra Leone, and in the city of Conakry, Guinea, we simulated realistic outbreak size distributions and compared them to reported outbreak sizes. These three empirical distributions lead to estimates for the proportion of detected spillover events and small outbreaks of 26% (range 8–40%, based on the full outbreak data), 48% (range 39–62%, based on the Sierra Leone data), and 17% (range 11–24%, based on the Guinea data). We conclude that at least half of all spillover events have failed to be reported since EVD was first recognized. We also estimate the probability of detecting outbreaks of different sizes, which is likely less than 10% for single-case spillover events. Comparing models of the observation process also suggests the probability of detecting an outbreak is not simply the cumulative probability of independently detecting any one individual. Rather, we find that any individual’s probability of detection is highly dependent upon the size of the cluster of cases. These findings highlight the importance of primary health care and local case management to detect and contain undetected early stage outbreaks at source.

## Author summary

Emerging infectious diseases are often not investigated in rural Africa unless outbreaks involve a sizeable number of cases. A number of different Ebola virus disease (EVD) outbreaks have been reported in the literature and in surveillance reports since its discovery in 1976. The majority of the reports are of large outbreaks. Given the low reported rate of transmission of Ebola, and the high frequency with which cases infect no one else, one might expect most outbreaks to be very small (<5 people). This is the first study to the authors’ knowledge that quantitatively estimates the number of undetected EVD outbreaks or probabilities of EVD outbreak detection by outbreak size. Although the total amount of evidence in this area is still limited, this study’s main result—that at least half of EVD outbreaks go undetected—is consistent under many different sets of assumptions. This is the most thorough estimation of EVD outbreak detection to date and corroborates the majority of more qualitative work on EVD surveillance, suggesting greater investment in primary health care and local surveillance will be important to detect EVD outbreaks early and consistently.

## Introduction

The last five years have seen an unprecedented number of cases of Ebola virus disease (EVD), which has taken an enormous toll in terms of mortality, economic damage, disruption to other public health programs and infrastructure, and public fear and mistrust [1–3]. Months of delay and dozens of cases may occur before an outbreak is reported, as in the 2013–2016 West African outbreak [4] and the most recent outbreak in the Democratic Republic of the Congo, which is ongoing at the time of writing [5–6]. This delay raises questions about how often and how early EVD outbreaks are detected, particularly those that lead to fewer cases.

We expect most EVD spillover events to be “dead ends” that do not transmit further, and most of these likely remain undetected. Spillover events from wildlife to people face myriad barriers to transmission and establishment—including host susceptibility, mobility, and onward contact with other susceptible individuals—often resulting in “stuttering chains” of transmission in people [7–8]. All diseases face the possibility of stochastic extinction upon introduction, and this is especially likely for a disease such as EVD that results in highly heterogeneous secondary transmission events [8–9]. Given a sufficiently skewed distribution of secondary infections, even a disease with a basic reproduction number (R_0_) greater than 1 is more likely than not to die out after a single index case [10]. However, few single-generation spillover events of EVD have been documented [11].

It is difficult to assess the efficiency of current health systems in detecting, treating, and preventing onward transmission of EVD, as the number of unobserved outbreaks is by definition unknown. Here we use three distinct data sets from the 2013–2016 outbreak in West Africa, using the properties of person-to-person EVD transmission to estimate the likely true distribution of EVD outbreak sizes. We do this by simulating the early stages of outbreaks and using maximum likelihood estimation of size-dependent detection rates to link them to the reported distribution of outbreak sizes. We thereby provide estimates for 1) the probability of observing an EVD outbreak of a given size and 2) the number of small outbreaks and spillover events that are likely to have gone undetected since EVD was first reported in 1976.

## Methods

### Ethics

We performed a secondary data analysis of several sources of data already in the public domain in anonymized form [9, 12–14]. We followed best practices guidelines for secondary data research issued by our institutional ethics committee, which indicated that no full ethical review was necessary.

### Analysis

Because published estimates of the true secondary case distribution of EVD vary widely—with estimates of R_0_ alone ranging from subcritical (i.e., <1) to >3 [15–16]—we parameterised our simulations with three previously estimated distributions, each based on different assumptions and data from different geographic areas. Each of these previous analyses provided parameter estimates and credible intervals for a negative binomial distribution. Negative binomial distributions are commonly used to represent secondary infections [8] and can be parametrised by the disease’s basic reproduction number (R_0_) and a dispersion parameter *k* measuring heterogeneity in secondary case numbers, with probability distribution $f(x)=\frac{\Gamma (x+k)}{\Gamma (k)x!}{\left(\frac{k}{k+R_{0}}\right)}^{k}{\left(1-\frac{k}{k+R_{0}}\right)}^{x}$. One set of estimates was obtained from all reported exposures from over 19,000 cases from Guinea, Liberia, and Sierra Leone (henceforth referred to as the full outbreak dataset) [12]. The second estimates were derived from cases in a single district of Sierra Leone, Western Area [13]. The final set of estimates was based on chains of transmission from 152 cases in early 2014 in Conakry, Guinea [9, 14].

**Fig 1:**
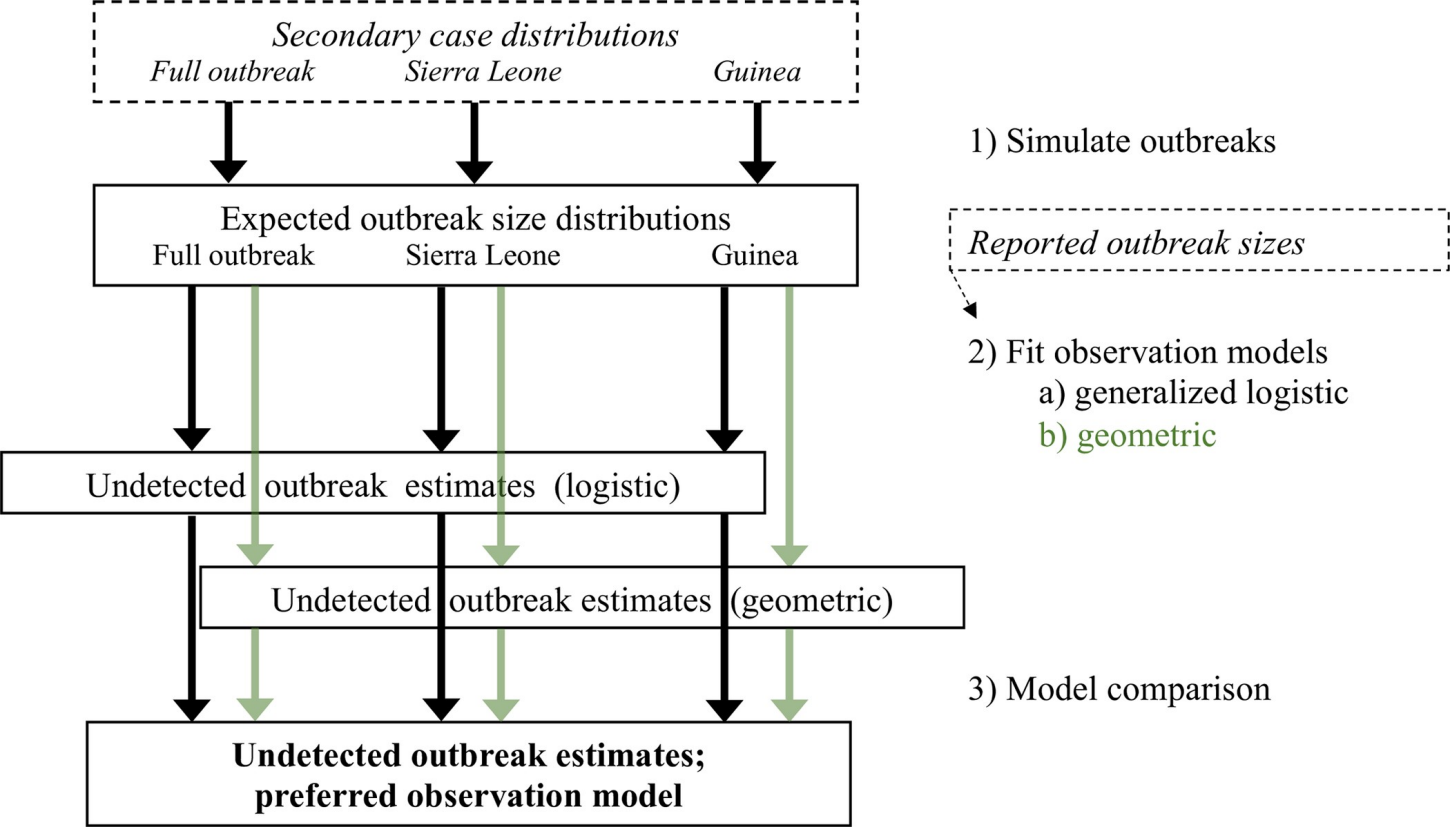
Diagram of stages of analysis (solid lines and numbered steps at right) and data inputs at each stage (italics and dashed lines). Parallel analyses were conducted for data from the full outbreak across West Africa, in Sierra Leone only (Western Area), and in Guinea only (Conakry).

A diagram of the full analysis is shown in Fig 1, and more information about the datasets and their resulting secondary infection distributions is presented in S1 Text.

For each dataset, we sampled 500 values each of R_0_ and the dispersion parameter *k* to approximate bounds supplied in the original papers (see S1 Text). From each parameter set, we simulated the early stages of each of 10^4^ outbreaks as stochastic branching processes for up to 50 generations or 57 cases (whichever limit was reached first). As expected, most outbreaks died out within a few generations, representing the stuttering chains of interest to our study. The 57-case threshold was chosen based on the following rationale. To allow us to link these simulated outbreaks to observed ones, we set a cutoff value based on two assumptions: 1) outbreaks larger than this cutoff are always observed, and 2) outbreaks smaller than this cutoff die out for primarily stochastic reasons, while larger outbreaks may die out for other reasons (such as interventions). Under the latter assumption, we fit our observation models according to the likelihood of any outbreak reaching a certain size rather than the (infinitesimally small) likelihood of large outbreaks dying out for purely stochastic reasons. After close inspection of the data, we set the initial cutoff to 57 cases to include the 1994 Gabon outbreak (52 cases), which was misidentified as a yellow fever outbreak while ongoing, and to exclude the 1996 Gabon outbreak (62 cases), which was subject to nosocomial control measures [17]. Our results are robust to the choice of cutoff value in a sensitivity analysis (S2 Fig).

We then modelled the probability Pr(*i*) of detecting an outbreak of size *i* using two possible linking functions: 1) the cumulative distribution function of the geometric distribution based on the probability *p* of detecting a single case, Pr(*i*) = 1−(1−*p*)^*i*^ (see e.g., [18]), and 2) a generalized logistic linking function (Pr(*i*) = (1+*e*^(*β−i*)^)^−*α*^). The observation function derived from the geometric distribution models assumes that the probability of detecting an outbreak is the cumulative probability of detecting at least one case, where each case has an equal and independent probability of being detected. The generalized logistic observation function allows that individual probability of detection to vary with outbreak size in a flexible way. We fit both distributions using the reported and expected (as generated above) distributions of outbreaks of 57 cases or smaller since 1976 using a coordinate descent method to iteratively select the expected numbers of outbreaks of each size and the parameters of the observation process (see S2 Text for details) [11]. We used corrected Akaike information criteria (AICc) to compare the two linking functions, then performed sensitivity analyses with the linking function with the lowest AICc values overall. We excluded from our distribution of reported outbreak sizes instances of laboratory infection and outbreaks of Reston virus, which follow extremely different spillover and transmission dynamics than African ebolaviruses (see S2 Table for full list of included outbreaks).

Finally, we performed several sensitivity and goodness-of-fit analyses. We assessed goodness of fit by simulating outbreaks and the observation process 10^4^ times. For each simulation, we sampled outbreak sizes and the observation process until 13 reported outbreaks smaller than the cutoff were detected (as per the data; see S2 Text). Our sensitivity analyses included variations in the cutoff value used to define outbreaks subject to stochastic extinction, a wider range of values of R_0_ and dispersion parameters, and the addition of a decreasing effective reproduction number with each generation of an emerging outbreak (e.g., due to control interventions).

## Results

**Fig 2:**
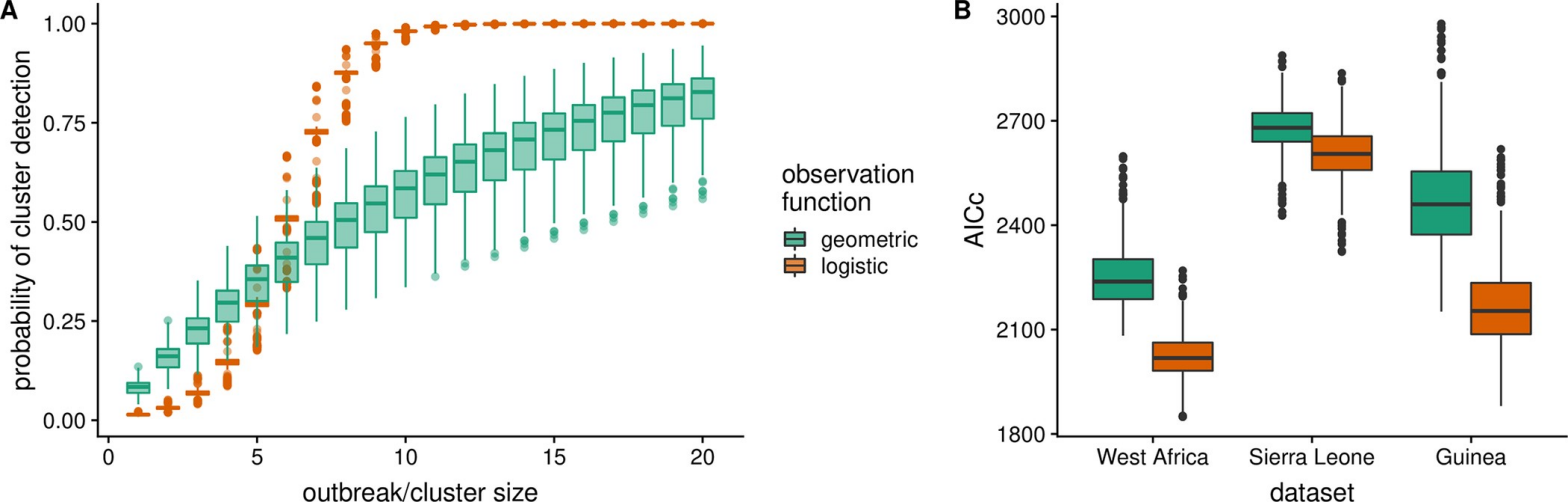
A. Estimated probabilities of cluster detection, by cluster size and observation function. The geometric observation function (green) is based on the cumulative distribution function of a geometric distribution (with a single parameter), while the logistic function (orange) is a generalized logistic function with two parameters. B. Ranges, 95% quantiles, interquartile ranges, and medians of AICc values for observation models applied to each dataset, by linking function used.

Parameter estimates for the generalized logistic observation function support a sigmoidal effect of cluster size on EVD detection probability. The geometric model fit to the full outbreak data produced a median individual probability of detection of 8.4% per case, irrespective of cluster size. In contrast, the logistic model predicts that an isolated case has a median of 2.4% probability of detection, while outbreaks of sizes greater than 10 approach 100% detection (Fig 2A). The generalized logistic model consistently outperformed the geometric model according to AICc (Fig 2B); all results below are from the generalized logistic model.

**Fig 3:**
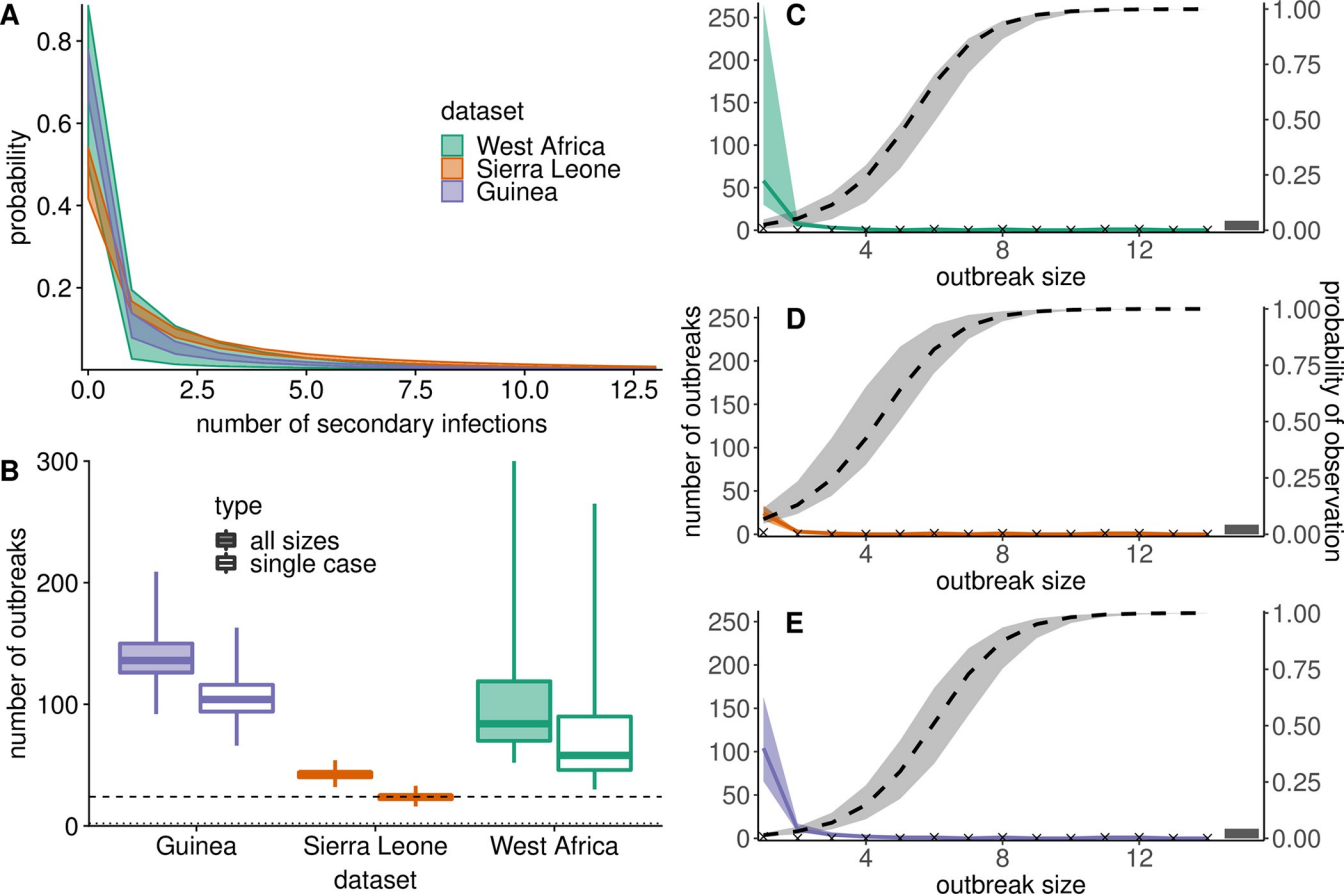
A. Ranges of secondary infection distributions of all three datasets. B. Estimated numbers of all small outbreaks and spillover events (solid) and single-case spillover events (white) for each dataset. The dashed line indicates all observed EVD outbreaks and the dotted line indicates all observed single-case spillover events. C-E. Medians and ranges of estimated true (observed and unobserved) small EVD outbreaks by outbreak size for the full West African outbreak (C), Western Area, Sierra Leone only (D), and Conakry, Guinea only (E). The colored areas indicate ranges of from all 500 parameter sets. The dashed lines and grey areas indicate estimated probabilities of observing an outbreak of each size (median and full ranges from 500 parameter sets). Points marked with ‘X’ indicate the number of observed outbreaks of each size. Outbreaks of 15 cases or larger are pooled.

For the full outbreak, Sierra Leona, and Guinea datasets, respectively, we estimate that medians of 67 (range 35–283; Fig 3C), 26 (range 15–37; Fig 3D), and 118.5 (range 75–192; Fig 3E) spillover events have gone undetected since EVD was first reported. These represent overall detection probabilities of 26.4% (range 7.8–40.7%), 48.0% (range 39.3–61.5%), and 16.8% (range 11.1–24.2%), respectively. Our model predicts that most of these undetected outbreaks were dead-end zoonotic spillovers causing a single human case. We estimate medians of 56 such undetected spillovers from the full outbreak data (range 28–263, corresponding to detection probabilities of 0.1–6.7%), 22 from the Sierra Leone data (range 14–31, corresponding to detection probabilities of 6.0–12.5%), and 101.5 from the Guinea data (range 64–161, corresponding to detection probabilities of 1.2–3.0%). Simulations of outbreak sizes and the observation process produce predicted observation counts concordant with the data (see S1 Fig).

Our results were consistent across several sensitivity analyses. Varying the outbreak size cutoff value from 5 cases to 55 cases (for the full outbreak data) only resulted in a total difference in estimated detection probabilities of about 8% of the minimum value (from 24.2% at a cutoff of 5 cases to 26.2% at 50 or 55 cases; see S2 Fig). We did not extend the range of considered values higher than our selected cutoff of 57 cases due to the sparseness of the data (e.g., there are no outbreaks between size 66 and size 124; see S2 Table) and the documented control efforts used to limit these outbreaks. Repeating our analysis for the full outbreak data across a wider range of plausible dispersion parameters and R_0_ values still produces detection probabilities in a similar range. The range of median estimates for the proportion of small outbreaks and spillover events within common existing estimates of R_0_ (1–1.5 by intervals of 0.25, inclusive) and *k* (0.1–0.6 by intervals of 0.25, inclusive) for EVD is 13%-45%. To estimate greater than 50% detection requires that R_0_ be >2, that *k* be >1.1, or that both R_0_>1.5 and *k*>0.85; all of these values are greater than most existing estimates (S3A Fig). Finally, we tested the effect on our results of allowing outbreaks to become slightly less infectious (e.g., due to successful attempts to control the outbreak, behavioural modifications, or pathogen evolution to become less virulent) by having R_eff_ decay 10, 20, or 30% per generation (S3B Fig). At very high values of the dispersion parameter (i.e., with minimal superspreading), the addition of this decay decreases the median proportion of detections. Within the same parameter ranges of common estimates (R_0_ 1–1.5; *k* 0.1–0.6, inclusive), median estimates for the detected proportion of small outbreaks and spillover events with R_eff_ decay of 0.3 range from 8% to 34%.

## Discussion

Our median estimates from all three datasets suggest at least half of all EVD spillover events (and possibly as many as 83%) have gone undetected. Although most of these spillover events have been ‘dead-ends’ or very small outbreaks, our models suggest this could represent well over 100 cases (from a minimum of 16 from the Sierra Leone data to a maximum of 317 from the full outbreak data). While the specific estimates of these missed small outbreaks are highly sensitive to assumptions about the underlying secondary case distribution, the central prediction is robust to different datasets, methodological choices such as the cutoff value for stochastic outbreaks, and a wide range of plausible R_0_ values and dispersion parameters.

We found consistently lower AICc values for the logistic observation model, which assumes a dependence of individual detection probability on cluster size. This suggests that outbreak surveillance is not adequately modelled as the combination of independent individual detection probabilities. We predict instead that individual cases are more likely to be correctly detected when part of a cluster of cases; identifying EVD cases that do not exist within clusters (or have not yet caused clusters) may be even more difficult to detect than previously assumed.

Across three datasets with very different estimates of the underlying offspring distribution (e.g., values of R_0_ from subcritical for the Guinea data to 2.4 for the Sierra Leone data), our analysis consistently predicts that most EVD spillover events and small outbreaks are not detected. That all three analyses from which we have drawn our estimates generate such different parameters highlights the difficulty of estimating them in a naïve, uncontrolled scenario when relevant data is collected in outbreak settings. However, the importance of superspreading is a consistent result of models applied to EVD, even at very early stages of outbreaks [19]; our sensitivity analysis (S3 Fig) predicts that at least 40% of spillover events are undetected as long as moderate superspreading occurs (*k*<1) and R_0_<2.

The true value of R_0_ for a typical spillover case is most likely lower than those used here, due to the unusual epidemiology of the West African outbreak [20]; the very low human population densities at which spillover events often occur [21–22]; and potential asymptomatic cases and uncharacteristic low-transmitting cases, which may not be sufficiently accounted for in existing R_0_ estimates [23]. Additionally, although asymptomatic infections are uncommon among contacts of human EVD cases [24], they may be a more common outcome of direct zoonotic spillover, e.g., with an ebolavirus strain that is poorly adapted to human hosts, as appears to be the case with Reston virus [7, 25]. Accounting for outbreaks that become less infectious over time (due to, e.g., control interventions, susceptible depletion, or host behavioural modification) causes our estimated outbreak detection rates to drop. We therefore expect that models combining our analysis with more accurate dynamics of EVD and its control would estimate lower detection rates than we present here. It is possible that all these factors render our results underestimations of the true frequency of spillover. If our underestimation is particularly extreme, it is possible hundreds or thousands of EVD spillovers have gone undetected, potentially explaining high seroprevalence of Ebola virus antibodies in some populations [26].

Due to these and other assumptions with less clear consequences, we intend this analysis not as a precise quantification of rates of EVD detection, but rather as a demonstration of the high probability that many spillovers go undetected and that many large outbreaks are not detected early. The regions from which our data come may be unrepresentative in ways we have not considered; no EVD spillovers have been reported in Sierra Leone, so the generalizability of the Sierra Leone dataset to the typical spillover case is unknown. Finally, we assume that each documented outbreak had a single index case from spillover. While we know of no outbreaks with multiple index cases, the origins of some have not been fully traced, and outbreaks originating from a multiple-spillover event are less likely to die out stochastically [27].

There is a clear need to improve outbreak detection and rapid response, and investment in these areas is among the most efficient ways of reducing EVD mortality [28]. Paving the way, Uganda instated a viral haemorrhagic fever surveillance programme in 2010 that has increased the number of outbreaks detected while reducing their mean size and mean time to confirmation [29]. We note that Uganda is one of only two countries to have detected spillovers resulting in a single case (S2 Table), which we expect to be the true most common outbreak size. This potential success does, however, highlight the lack of spatial resolution as a limitation of our study. The small number of total observed EVD spillover events means this analysis is uninformative on a country-by-country basis, obscuring potential differences in detection between countries. Additional work could consider more systematically spatial variation in contexts likely to lead to spillover and onward transmission, as well as barriers to treatment and reporting.

Our estimates suggest that most spillover events and small outbreaks of EVD are not reported to international bodies but rather are handled locally, likely as fevers of unknown origin or mischaracterised as more common causes of fever (e.g., malaria) [30]. Supporting core public health and sanitation infrastructure in the areas where spillover is likely to occur may prove vital to preventing the onward transmission of these unseen index cases. Furthermore, promoting the safe management of fever and enhancing local diagnostic capacity has the potential to improve facility-based national surveillance systems and ultimately increase the chance of early detection of EVD outbreaks, both large and small.

## Supporting information

S1 Text Data sources and secondary infection distributions. (PDF)

S2 Text Estimating observation rates and unobserved outbreaks. (PDF)

S3 Text Sensitivity of results to outbreak size cutoff value. (PDF)

S4 Text Index case scenarios. (PDF)

S1 Table Parameters and estimates by data source. (PDF)

S2 Table Reported outbreaks included in analysis, sorted by size. (PDF)

S1 Fig Simulated outbreak observations. Predicted numbers of total outbreaks (green) and observed outbreaks (black) of each size from 10^4^ simulations of the models fit to the full outbreak data. Lighter regions and darker regions represent the 95% CI and IQR of simulations, respectively. Points marked with X represent real reported outbreaks.
(TIF)

S2 Fig Effects of outbreak cutoff size on detection estimates. Medians, 95% confidence intervals, and interquartile ranges for estimated proportions of all EVD outbreaks to have been detected vs. the cutoff value for stochastic outbreaks chosen for this analysis (based on the full outbreak data). Points marked with X along the bottom axis indicate the sizes of real reported outbreaks.
(TIF)

S3 Fig Effects of R_0_ value, dispersion parameter, and R_eff_ decay on detection estimates. Estimated proportion of all EVD outbreaks observed as a function of R_0_ and dispersion parameter *k*, where higher values of *k* indicate greater heterogeneity in secondary infections. Each subplot was generated with a different decay constant (δ) that reduces the effective reproductive number R_eff_ by 0, 10, 20, or 30% per generation. All estimations were performed with a cutoff of 57 cases. The area indicated by the black box represents parameter values included in the main analysis, while the white lines indicate estimated 50% contours.
(TIF)
