# Supplementary material for: Estimating undetected Ebola spillovers
Source: PLoS Negl Trop Dis. 2019 Jun 13;13(6):e0007428. doi: 10.1371/journal.pntd.0007428 (PMC6563953; doi:10.1371/journal.pntd.0007428)
Supplement: S1 Text — (PDF) [file pntd.0007428.s001.pdf]

**Estimating undetected Ebola spillovers: Supplementary material**
Emma E Glennon, Freya L Jephcott, Olivier Restif, James L N Wood

## S1 Text. Data sources and secondary infection distributions

All three sets of secondary infection distribution estimates were based on data from the large EVD outbreak in West Africa in 2013-2016, although they accounted for different data types, time periods, locations, and assumptions about transmission.

The full outbreak data is based on the largest dataset of the three: approximately 33% of the more than 19,000 cases who reported exposures during the course of outbreak response [1]. The authors of the study reconstructed transmission networks from these reported exposures; they estimated the offspring distribution from fit models of the out-degree distribution of the network under varying assumptions of the relationship of the sampled network to the full outbreak. They produced two estimates of $R_0$ and $k$ under more and less conservative assumptions about that relationship, and we used the parameters of these two estimates as the endpoints for sampling. Advantages of this dataset for our analysis include the amount of directly applicable exposure data, a large and diverse geographic area that may be more representative of data from urban areas alone, and the thorough analysis of the underlying exposure network. The primary disadvantage of this dataset is that it uses data from up through May 2015, by which time the outbreak had been ongoing for over a year and extensive control measures and behavioral modifications were in place.

The other two sets of offspring distribution estimates—from Western Area, Sierra Leone [2], and Conakry, Guinea [3-4]—may more closely approximate the transmission processes associated with spillover/index cases for various reasons. The Guinea data represents very early stages of the outbreak (up to August 2014), but it consists of cases in a large urban center, in a country with a lower-than-average $R_0$ [5], and its estimation methods are quite simple. The analysis applied to the Sierra Leone data analyzes changes in the offspring distribution across stages of the outbreak and across age classes, thereby offering insights into the process of superspreading at early stages of outbreaks; however, much of this data also originated in an urban center (Freetown), in a country with a high estimate of $R_0$ compared to most of the same outbreak [5], and it includes only data from safe burials although unsafe burials have been estimated to transmit to as many as 6.1 additional cases per death [6]. For these reasons, we believe the Sierra Leone dataset represents a conservative estimate of the amount of superspreading, while the Guinea dataset provides a less conservative estimate; parameter estimates from the full outbreak dataset span a wide range, but their median values fall in between those from Sierra Leone and Guinea.

For all datasets, we assumed independence of $R_0$ and the dispersion parameter. This assumption may have produced a slightly wider range of possible distributions than in the original paper. Table S1 shows results from all three sets of parameters.

[1] International Ebola Response Team, Agua-Agum J, Ariyarajah A, Aylward B, Bawo L, Bilivogui, P, et al. Exposure patterns driving Ebola transmission in West Africa: A retrospective observational study. PLoS Med. 2016;13(11):1–23.

[2] Lau MSY, Dalziel BD, Funk S, McClelland A, Tiffany A, Riley S, et al. Spatial and temporal dynamics of superspreading events in the 2014–2015 West Africa Ebola epidemic. Proc Nat Acad Sci. 2017;114(9):2337–2342.
[3] Althaus CL. Ebola superspreading. Lancet Inf Dis. 2015;15(5):507–508.
[4] Faye O, Boëlle PY, Heleze E, Faye O, Loucoubar C, Magassouba N, et al. Chains of transmission and control of Ebola virus disease in Conakry, Guinea, in 2014: An observational study. Lancet Inf Dis. 2015;15(3):320–326.
[5] Krauer F, Gsteiger S, Low N, Hansen CH, Althaus CL. Heterogeneity in District-Level Transmission of Ebola Virus Disease during the 2013-2015 Epidemic in West Africa. PLoS Negl Trop Dis. 2016;10(7).
[6] Drake JM, Kaul RRB, Alexander LW, O'Regan SM, Kramer AM, Pulliam JT, et al. Ebola cases and health system demand in Liberia. PLoS Biol. 2015;13(1).
