# Supplementary material for: Estimating undetected Ebola spillovers
Source: PLoS Negl Trop Dis. 2019 Jun 13;13(6):e0007428. doi: 10.1371/journal.pntd.0007428 (PMC6563953; doi:10.1371/journal.pntd.0007428)
Supplement: S2 Text — (PDF) [file pntd.0007428.s002.pdf]

Estimating undetected Ebola spillovers: Supplementary material
Emma E Glennon, Freya L Jephcott, Olivier Restif, James L N Wood

S2 Text. Estimating observation rates and unobserved outbreaks

Likelihood function.

We fit a function linking the simulated distribution of outbreak sizes to the reported number of outbreaks of each size (Table S2), corresponding to a size-dependent probability of observation. This function was modelled in two ways: 1) the geometric cumulative distribution function of outbreak size *i* ($\Pr(i) = 1 - (1-p)^i$), a model that assumes all individuals have equal and independent probabilities of being detected; and 2) as a generalized logistic function of outbreak size *i* ($\Pr(i) = (1 + e^{(\beta - i)})^{-\alpha}$). The generalized logistic linking function can be thought of as a geometric function where the value of p can change flexibly with i; i.e., the chance of detecting any one case in a cluster of size i is $1 - \sqrt[i]{1 - (1 + e^{\beta - i})^{-\alpha}}$. The likelihood for either linking function was:

$$\mathcal{L}(N|\theta) = T_a! * \prod_{i=1,2\ldots 57,58+} \frac{f_i^{T_i}}{T_i!} * \prod_{i=1}^{57} \binom{T_i}{N_i} \Pr(i)^{N_i} (1 - \Pr(i))^{T_i - N_i}$$

Where θ is either p or {α, β}, N_i and T_i are the observed and expected numbers of outbreaks of size *i*, respectively (with T_{58+} as the number of all outbreaks larger than the cutoff), T_a is the total number of observed and unobserved outbreaks, and f_i is the density of outbreaks of size *i* from 10^4 outbreak simulations (with densities of zero set to the minimum nonzero density). The two components of the likelihood function represent 1) $T_a! * \prod_{i=1,2\ldots 57,58+} \frac{f_i^{T_i}}{T_i!}$: the likelihood of T_i outbreaks of size *i* being drawn from T_a outbreaks given the outbreak size distribution generated in simulation and 2) $\prod_{i=1}^{57} \binom{T_i}{N_i} \Pr(i)^{N_i}(1 - \Pr(i))^{T_i - N_i}$: the likelihood of observing N_i outbreaks of size *i* given T_i true outbreaks of size *i* and some probability of observing an outbreak of size *i* (Pr(*i*)).

Likelihood estimation by coordinate descent.

Because $T=\{T_1, T_2 \ldots T_{57}, T_{58+}\}$ is unobserved, we maximized this likelihood following a block coordinate descent method that iteratively optimizes T and θ. This method is similar to the expectation maximization (EM) meta-algorithm, which is commonly used to fit models with latent variables; however, due to computational constraints we optimize T at each iteration based on steepest descent and perturbation of local (negative log) likelihood minima rather than considering the expectation of all possible combinations of 58 latent variables. At each of 1000 iterations (or until a tolerance in the difference in likelihood was reached), we maximized the likelihood as follows:

1. Setting a starting estimate for the true number of outbreaks (T_a) as round($^{T_{58+}}/_{f_{58+}}$);
2. Setting a starting estimate for T as the medians of the binomial distributions of size T_a and probabilities f;
3. Setting minimum values of each value of T_i as the observed number of outbreaks of size *i* and a minimum value of T_a as the observed number of all outbreaks;

4. ***T* selection (expectation step analogue).** Finding an estimate of T ($\hat{T}$) that maximizes the likelihood given a fixed estimate of θ:
    a. Calculating the changes to the likelihood function of all single-outbreak increases or decreases in any member of $\hat{T}$ that satisfy the minima set in step 3;
    b. For each possible single-outbreak increase or decrease in $\hat{T}$, performing the increment or decrement that most maximizes the likelihood, then updating $\hat{T}_a$ accordingly;
    c. Repeating steps a and b until a local likelihood maximum is reached;
    d. To prevent returning local maxima, perturbing $\hat{T}$ 2000 times at each of 10 perturbation strengths.
    e. Updating $\hat{T}$ and returning to step a if the likelihood of any perturbed $\hat{T}$ is higher than the current maximum likelihood estimate.
5. **θ selection (maximization step).** Maximizing the likelihood over θ given $\hat{T}$, using the Nelder-Mead algorithm for the logistic observation function or Brent optimization for the geometric observation function.
6. Returning to step 4 and iterating until no changes improve the (negative log) likelihood more than the tolerance threshold of 10^{-10} or 1000 iterations have occurred.

Finally, to test the globality of our maximum likelihood estimates, we performed additional perturbations for a random subset of 150 the final 1500 estimates. We perturbed these estimates of $\hat{T}$ for an additional 4000 perturbations at each of 500 strengths. None of these perturbations resulted in higher likelihood estimates. We calculated AICc values for all final likelihood estimates and compared them across observation models; models with the geometric observation function consistently resulted in lower AICc values.

Goodness of fit.

To confirm the goodness of fit of the final models, we simulated the outbreak observation process 10^4 times. For each of these simulations, we:

1) Sampled a distribution of outbreak sizes from simulation, based on the full outbreak dataset parameters,
2) Sampled an outbreak size (i) with weights from the simulated distribution,
3) Randomly assigned each outbreak to be reported or unreported, according to the corresponding estimate of $\Pr(i)$ from the fit (logistic) observation model, and
4) Repeated steps 2 and 3 until a total of 13 outbreaks were observed.

Fig S2 shows the results of these simulations.
