# Supplementary material for: Estimating undetected Ebola spillovers
Source: PLoS Negl Trop Dis. 2019 Jun 13;13(6):e0007428. doi: 10.1371/journal.pntd.0007428 (PMC6563953; doi:10.1371/journal.pntd.0007428)
Supplement: S3 Text — (PDF) [file pntd.0007428.s003.pdf]

Estimating undetected Ebola spillovers: Supplementary material
Emma E Glennon, Freya L Jephcott, Olivier Restif, James L N Wood

S3 Text. Sensitivity of results to outbreak size cutoff value

The cutoff size used in this work differentiates outbreaks subject to largely stochastic dynamics (i.e., heterogeneity in transmission) from those subject to largely deterministic dynamics (i.e., following the trajectory expected for a pathogen with $R_0>1$) and control. This cutoff is necessary to decide which outbreaks are considered by which term within our likelihood function. We chose a cutoff for outbreaks considered stochastic based on the histories of observed outbreaks; we set the cutoff midway between the 1994 and 1996 outbreaks in Gabon (52 and 62 outbreaks, respectively). The former outbreak was initially mischaracterised as a yellow fever outbreak and only retrospectively identified as EVD, so it was not subject to EVD-specific control efforts, while the latter outbreak was identified as EVD approximately 3 months before the final confirmed case (5 months before the outbreak was officially declared over) [1].

This choice has a minimal effect on our analysis (Fig. S2). We repeated the analysis of the full outbreak data across a wide range of cutoff values (every 5 cases from 5 to 55 cases) and found the choice of cutoff value resulted in a maximum difference of 2% (from 24.2% probability of detection at a cutoff of 5 cases to 26.2% probability at 50 or 55 cases).

[1] Georges A, Leroy EM, Renaut AA, Benissan CT, Nabias RJ, Ngoc MT, et al. Ebola Hemorrhagic Fever Outbreaks in Gabon, 1994–1997: Epidemiologic and Health Control Issues. J Inf Dis. 1999;179:S65–S75.
