# Supplementary material for: Estimating undetected Ebola spillovers
Source: PLoS Negl Trop Dis. 2019 Jun 13;13(6):e0007428. doi: 10.1371/journal.pntd.0007428 (PMC6563953; doi:10.1371/journal.pntd.0007428)
Supplement: S4 Text — (PDF) [file pntd.0007428.s004.pdf]

Estimating undetected Ebola spillovers: Supplementary material
Emma E Glennon, Freya L Jephcott, Olivier Restif, James L N Wood

S4 Text. Index case scenarios

A key assumption of this work is that index cases infected directly from zoonotic spillover are equally likely to transmit to other people as cases in naïve outbreak scenarios. Evidence for or against this assumption in the case of EVD is limited, although some zoonoses may be less likely to transmit from index cases, e.g., due to host barriers the pathogen must evolve to overcome or due to a lack of control efforts. We tested the consequences of this assumption by 1) expanding the range of allowed R_0 values for early-stage epidemics to include both slightly subcritical infection (R_0=0.5 or 0.75) and greater infectivity than estimated for the West African outbreak (R_0 up to 3), 2) expanding the range of dispersion parameters considered to include lower amounts of superspreading (*k* up to 2.1), and 3) simulating the effects of control interventions and behavior change by producing sets of outbreak size simulations with R_{eff} decay of 10, 20, or 30% per generation. We simulated 10^4 outbreaks each with values of R_0 from 0.5 to 3 at intervals of 0.25, values of *k* from 0.1 to 2.1 at intervals of 0.25, and values of R_{eff} decay of 0 to 30% per generation, at intervals of 10%. The results from this analysis are presented in Fig. S3.

Estimates across this wide range of scenarios result in some variation, but the conclusion that the majority of EVD outbreaks are undetected remains except when R_0>2 and *k*>1. Addition of a decay constant in general results in lower estimates of observation probabilities. This suggests that our estimates of undetected outbreaks are more likely biased toward underestimation than overestimation by not accounting for control efforts or susceptible depletion.
