# Supplementary material for: Estimating undetected Ebola spillovers
Source: PLoS Negl Trop Dis. 2019 Jun 13;13(6):e0007428. doi: 10.1371/journal.pntd.0007428 (PMC6563953; doi:10.1371/journal.pntd.0007428)
Supplement: S1 Table — (PDF) [file pntd.0007428.s005.pdf]

S1 Table. Parameters and estimates by data source. Estimates from analysis based on published secondary infection distributions based on data from across West Africa [1], from Western Area, Sierra Leone [2], and from Conakry, Guinea [3-4].

	Full outbreak	Sierra Leone	Guinea
**R_0**	~unif(1, 1.5)	~logNormal(ln(2.39),0 .09)	~logNormal(ln(0.95),0.09)
**Dispersion (k)**	~unif(0.03,0.52)	~normal(0.37, 0.025)	~normal(0.18, 0.025)
**Median number of undetected outbreaks since 1976 (range)**	67 (35-283)	26 (15-37)	118.5 (75-192)
**Median number of undetected 'dead-end' spillovers since 1976 (range)**	56 (28-263)	22 (14-31)	101.5 (64-161)
**Median outbreak detection probability (range)**	26.4% (7.8-40.7%)	48.0% (39.3-61.5%)	16.8% (11.1-24.2%)
**Median 'dead-end" spillover detection probability (range)**	3.4% (0.1-6.7%)	8.3% (6.0-12.5%)	1.9% (1.2-3.0%)

[1] International Ebola Response Team, Agua-Agum J, Ariyarajah A, Aylward B, Bawo L, Bilivogui, P, et al. Exposure patterns driving Ebola transmission in West Africa: A retrospective observational study. PLoS Med. 2016;13(11):1–23.
[2] Lau MSY, Dalziel BD, Funk S, McClelland A, Tiffany A, Riley S, et al. Spatial and temporal dynamics of superspreading events in the 2014–2015 West Africa Ebola epidemic. Proc Nat Acad Sci. 2017;114(9):2337–2342.
[3] Althaus CL. Ebola superspreading. Lancet Inf Dis. 2015;15(5):507–508.
[4] Faye O, Boëlle PY, Heleze E, Faye O, Loucoubar C, Magassouba N, et al. Chains of transmission and control of Ebola virus disease in Conakry, Guinea, in 2014: An observational study. Lancet Inf Dis. 2015;15(3):320–326.
