# Supplementary material for: Estimating undetected Ebola spillovers
Source: PLoS Negl Trop Dis. 2019 Jun 13;13(6):e0007428. doi: 10.1371/journal.pntd.0007428 (PMC6563953; doi:10.1371/journal.pntd.0007428)
Supplement: S2 Table — (PDF) [file pntd.0007428.s006.pdf]

S2 Table. Reported outbreaks included in analysis, sorted by size. Outbreaks reported by multiple countries but originating from the same spillover event (e.g., in the case of the 2013-2016 West African outbreak) are pooled. Outbreaks occurring outside of Africa, in laboratory settings, or of Reston virus are excluded. A 1994 single-case spillover of Taï Forest virus disease in a researcher infected in Cote d'Ivoire but treated in Switzerland was also excluded. All outbreak information is used as provided by the Centers for Disease Control and Prevention [1].

Year of origin	Country of origin	Other countries affected	Cases
1977	Democratic Republic of the Congo		1
2011	Uganda		1
2012	Uganda		6
2017	Democratic Republic of the Congo		8
2012	Uganda		11
2005	Republic of the Congo		12
2004	Sudan		17
2008	Democratic Republic of the Congo		32
1979	Sudan		34
2003	Republic of the Congo		35
2012	Democratic Republic of the Congo		36
1996	Gabon		37
1994	Gabon		52
1996	Gabon	South Africa	62
2014	Democratic Republic of the Congo		66
2001	Gabon and Republic of the Congo		124
2007	Uganda		131
2002	Republic of the Congo		143
2007	Democratic Republic of the Congo		264
1976	Sudan		284
1995	Democratic Republic of the Congo		315
1976	Democratic Republic of the Congo		318
2000	Uganda		425
2013	Guinea	Liberia, Sierra Leone, Italy, Mali, Nigeria, Senegal, Spain, United States	28645

[1] Centers for Disease Control and Prevention. Years of Ebola virus disease outbreaks [Internet]. Atlanta: 2018. Available from: http://www.cdc.gov/vhf/ebola/outbreaks/history/chronology.html.
