# Supplementary figures and images for: Estimating undetected Ebola spillovers
Source: PLoS Negl Trop Dis. 2019 Jun 13;13(6):e0007428. doi: 10.1371/journal.pntd.0007428 (PMC6563953; doi:10.1371/journal.pntd.0007428)

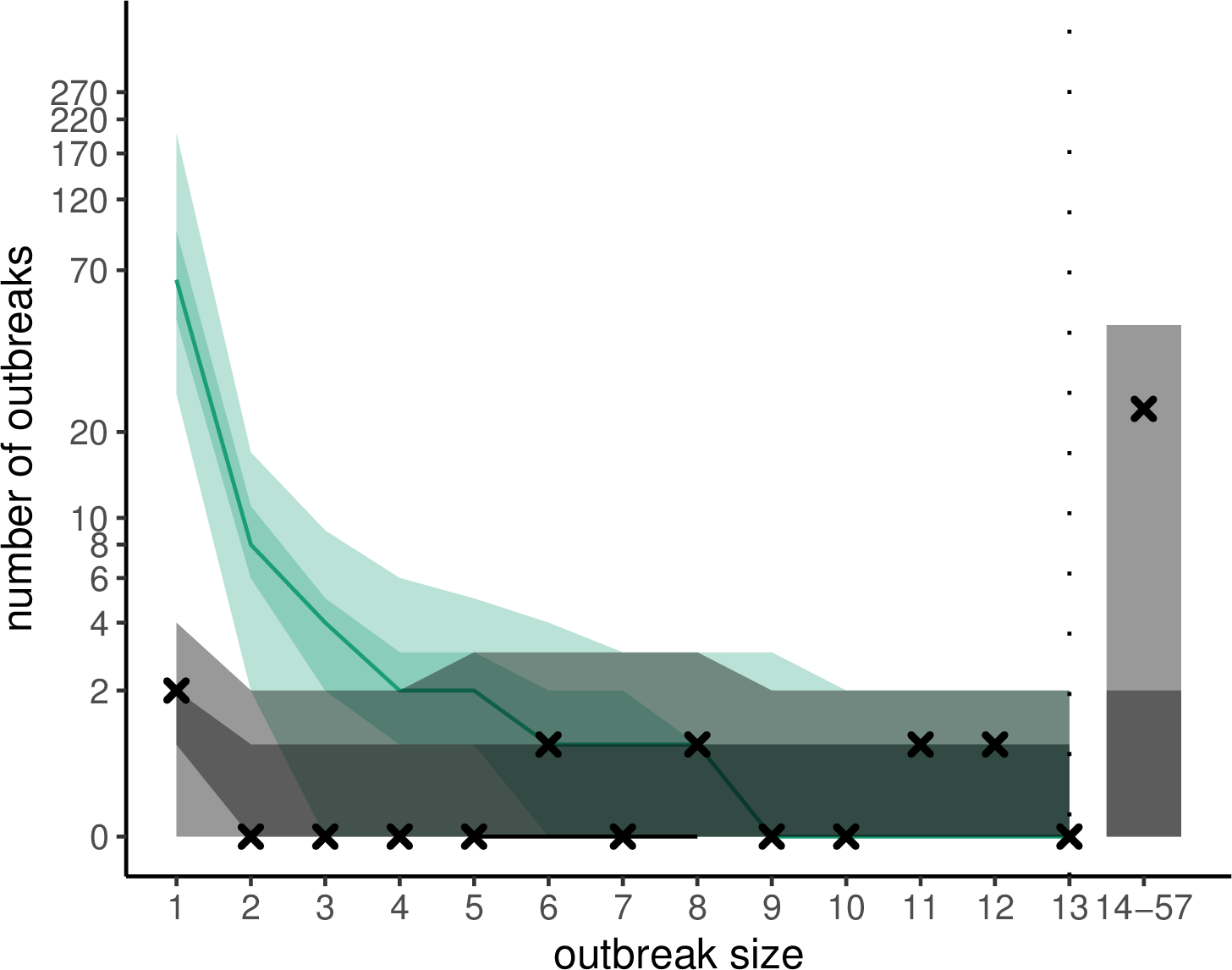

Supplement: S1 Fig — Predicted numbers of total outbreaks (green) and observed outbreaks (black) of each size from 104 simulations of the models fit to the full outbreak data. Lighter regions and darker regions represent the 95% CI and IQR of simulations, respectively. Points marked with X represent real reported outbreaks. (TIF) [file pntd.0007428.s007.tif]

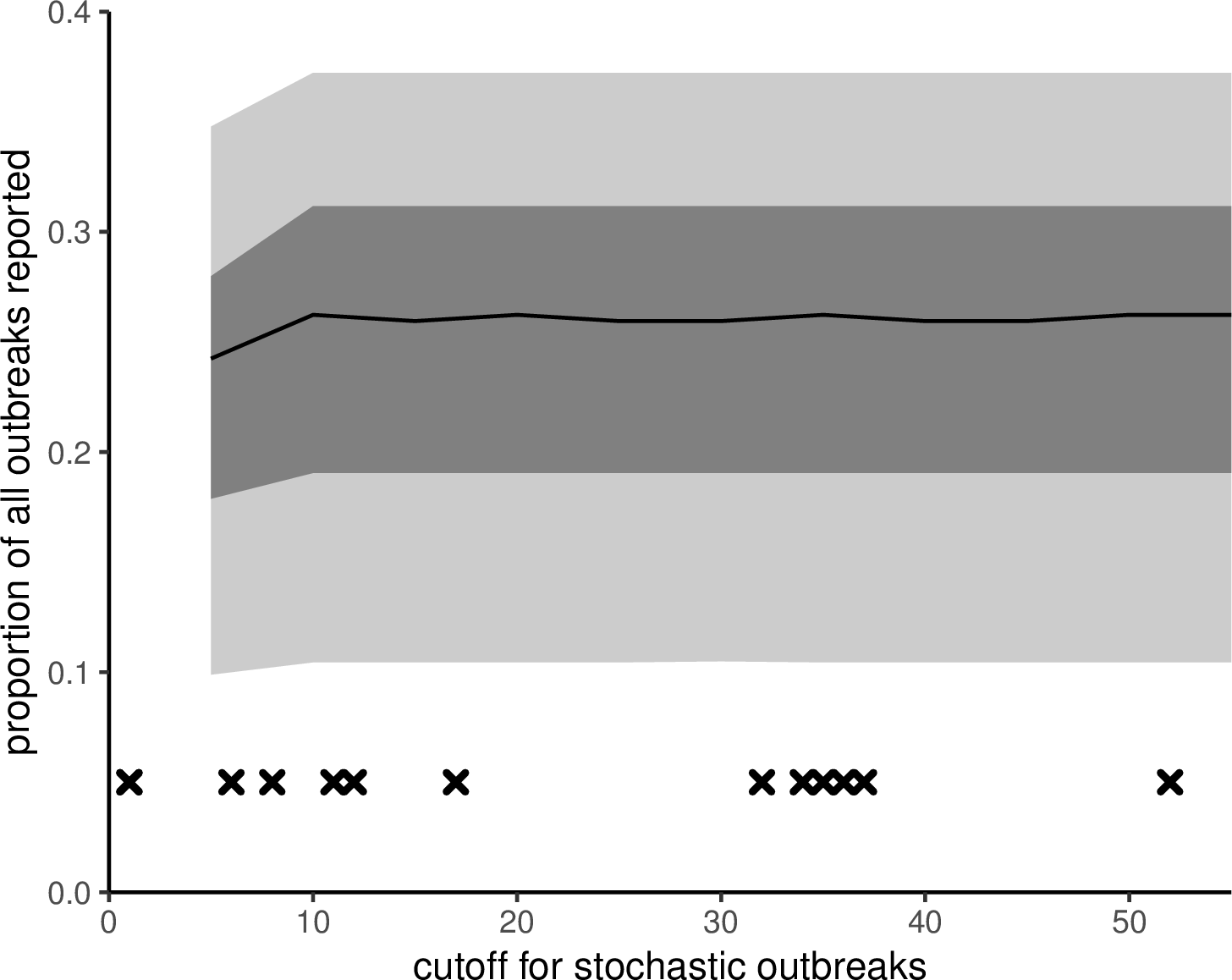

Supplement: S2 Fig — Medians, 95% confidence intervals, and interquartile ranges for estimated proportions of all EVD outbreaks to have been detected vs. the cutoff value for stochastic outbreaks chosen for this analysis (based on the full outbreak data). Points marked with X along the bottom axis indicate the sizes of real reported outbreaks. (TIF) [file pntd.0007428.s008.tif]

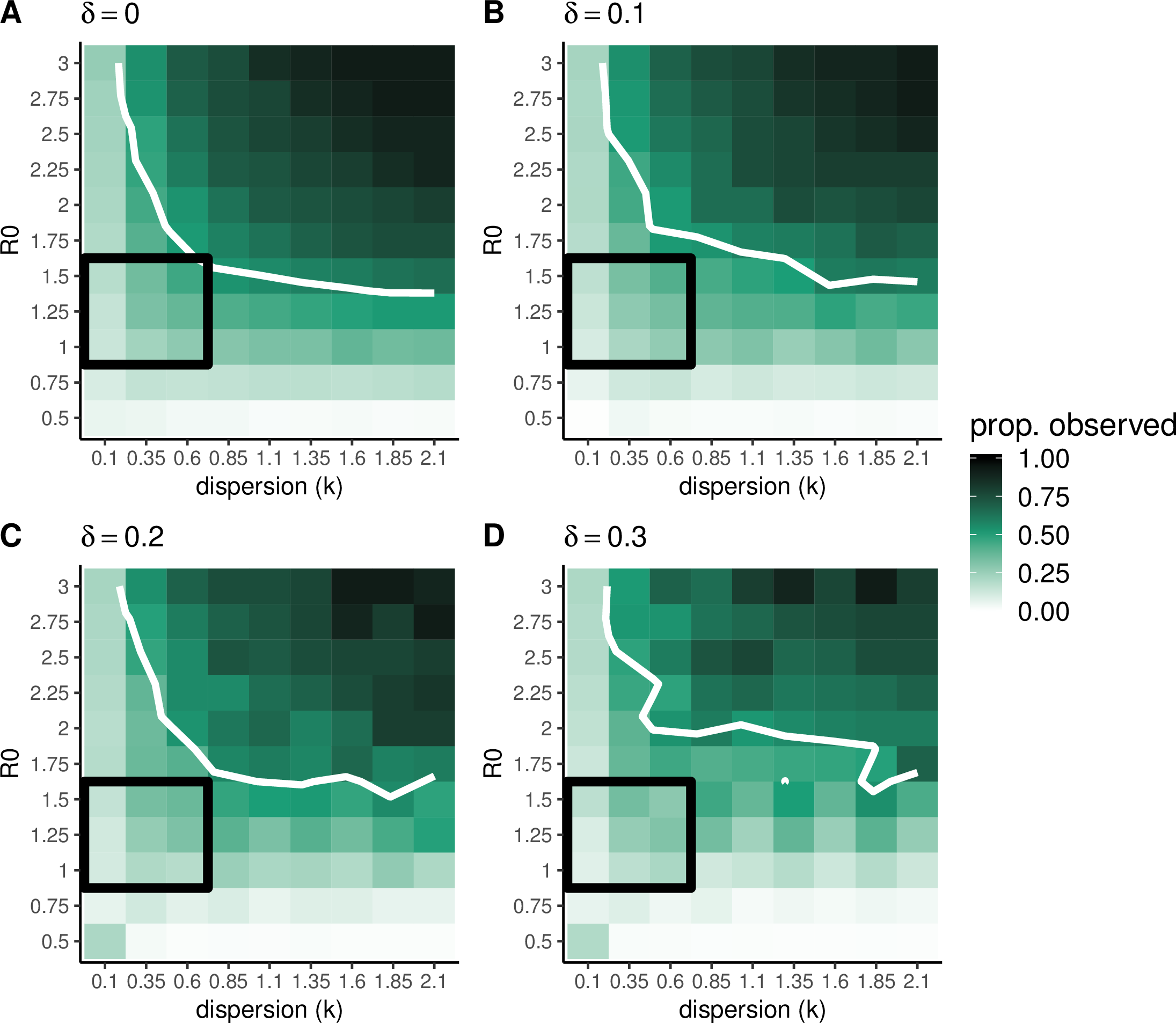

Supplement: S3 Fig — Estimated proportion of all EVD outbreaks observed as a function of R0 and dispersion parameter k, where higher values of k indicate greater heterogeneity in secondary infections. Each subplot was generated with a different decay constant (δ) that reduces the effective reproductive number Reff by 0, 10, 20, or 30% per generation. All estimations were performed with a cutoff of 57 cases. The area indicated by the black box represents parameter values included in the main analysis, while the white lines indicate estimated 50% contours. (TIF) [file pntd.0007428.s009.tif]
